# High rate of core promoter and precore mutations in patients with chronic hepatitis B

**DOI:** 10.1007/s12072-014-9598-5

**Published:** 2014-12-25

**Authors:** Sumbella F. Baqai, James Proudfoot, Debbie H. Yi, Michael Mangahas, Robert G. Gish

**Affiliations:** 1 Department of Internal Medicine, Alameda County Medical Center, 1411 E. 31st St., Oakland, CA 94602-1018 USA; 2 Biostatistics Unit, Clinical and Translational Research Institute, University of California, San Diego, 9500 Gilman Drive, La Jolla, CA 92093 USA; 3 Emergency Medicine and Neurocritical Care, Hospital of the University of Pennsylvania, 3400 Spruce St, Philadelphia, PA 19104 USA; 4 University of California, San Francisco/University of California, Berkeley, Joint Medical Program, University of California, San Francisco, School of Medicine, 513 Parnassus Avenue, San Francisco, CA 94143 USA; 5 Robert G. Gish Consultants, LLC, 6022 La Jolla Mesa Drive, San Diego, CA 92037 USA; 6 Department of Medicine, Division of Gastroenterology and Hepatology, Stanford University, 291 Campus Drive, Stanford, CA 94305 USA; 7 Hepatitis B Foundation, 3805 Old Easton Rd, Doylestown, PA 18902 USA; 8 St. Joseph’s Hospital and Medical Center, 350 W Thomas Road, Phoenix, AZ 85013 USA; 9 Department of Clinical Medicine, University of Nevada School of Medicine, 2040 West Charleston Boulevard, Las Vegas, NV 89102 USA; 10 Hepatitis B Foundation, 6022 La Jolla Mesa Drive, San Diego, CA 92037 USA

**Keywords:** Chronic hepatitis B, Precore mutations, Core promoter mutations, HBeAg-positive, HBeAg-negative, ALT

## Abstract

**Background:**

The prevalence of precore (PC) and core promoter (CP) mutations in patients with chronic hepatitis B virus (HBV) infection (CHB) and their impact on liver disease is incompletely defined in the United States.

**Methods:**

A retrospective chart review using a cross-sectional approach of 1,186 CHB patients was conducted.

**Results:**

Of 926 patients tested for HBV e antigen (HBeAg), 37 % were HBeAg+. Of 194 patients tested for mutations, 80 % had PC or CP mutations or both; 89 % of HBeAg-negative and 56 % of HBeAg+ patients had PC or CP mutations or both (*p* < 0.001). The mean log_10_ ALT was significantly lower in patients with both mutations compared to patients without mutations. The mean log_10_ HBV DNA was significantly lower in patients with only PC mutations (4.82) compared to patients without mutations (5.71, *p* = 0.019). With the study population divided into four subgroups based on ALT level at time of diagnosis, cirrhosis incidence was significantly higher in patients with ALT 1–2 × ULN and ALT > 2 × ULN compared to patients with ALT ≤ 0.5 × ULN.

**Conclusions:**

Our finding that PC and CP mutations may be associated with milder liver disease in some patients could serve as the basis for longitudinal studies to help delineate treatment need and duration in patients with these mutations. If confirmed, the finding of an association between ALT 1–2 × ULN and increased incidence of cirrhosis could call into question guidelines which only recommend treatment with ALT > 2 × ULN.

## Introduction

Chronic hepatitis B (CHB) patients with hepatitis B e antigen-negative (HBeAg-negative) disease often have mutations in the core promoter (CP) and precore (PC) regions of hepatitis B virus (HBV) that explain the decreased HBeAg production [1]. Many HBeAg-positive patients have infection that includes both wild-type virus and variants with CP mutations [2, 3] and, less commonly, PC mutations [2, 4]. In patients with ongoing viral replication, seroconversion, HBeAg loss, and development of the hepatitis B e antibody (anti-HBe) are often associated with emergence and selection of replication-competent virions with PC mutations [3]. In HBeAg+ patients the progression toward an “eAg-negative” disease may begin with CP mutations (and, to a lesser extent, PC mutations) well before the date of ultimate HBeAg loss.

A 2003 US study reported that the prevalence of PC and CP variants was much more common in HBeAg-negative than in HBeAg-positive patients (PC, 38 vs. 9 %; CP, 51 vs. 36 %) but inclusion of only treatment-naïve patients may have resulted in prevalence underestimation [4]. A 2013 US study of HBeAg+ patients reported a prevalence of 28 % (PC mutations) and 17 % (CP mutations); however, the patient population was almost entirely (92.3 %) Asian Americans with genotypes B and C [5]. Thus, the baseline US prevalence of PC and CP mutants in community settings that include both treatment-naïve and -experienced patients from all ethnic backgrounds remains undefined.

There is conflicting data on the effect of PC and CP variants on the risk of serious liver disease. Multiple studies have reported an association between CP mutations and increased liver inflammation [6, 7], cirrhosis [8] and hepatocellular carcinoma (HCC) [8–13]. Some researchers have reported an association between PC mutations and fulminant hepatitis [14, 15], while others found either no such association [16] or, in fact, an association with decreased inflammation [6]. One study reported an association between PC variant G1896A and a decreased risk of HCC [13]. Furthermore, various studies have reported (1) no correlation between PC/CP mutations and HBV DNA levels [17], (2) an association between most CP and PC mutations with significantly reduced viral replication [18], (3) an association between up-regulation of viral replication with CP mutations other than those at 1762/1764 [19], and (4) an association between increased viral replication and the PC mutation [20].

We describe here a large cross-sectional study of both HBeAg+ and HBeAg− negative CHB patients from community settings, including patients from all ethnic backgrounds, in which we evaluated the presence of PC and CP mutations (with separate analyses of treatment-naïve and -experienced patients), and the possible association of these mutations with ALT levels, HBV DNA, prevalence of cirrhosis and incidence of HCC. We also assessed the association of various levels of ALT at the time of diagnosis with the incidence of cirrhosis and HCC.

## Methods

A total of 1,186 CHB patients who had undergone long-term follow up in 14 liver clinics in California and Nevada linked to California Pacific Medical Center (CPMC) were retrospectively analyzed using a cross-sectional approach; the CPMC institutional review board approved the study. All patients seropositive for hepatitis B surface antigen (HBsAg) and hepatitis B core antibody (anti-HBc) immunoglobulin G (IgG) for at least 6 months were included. Patient demographics (age, sex, country of origin, ethnicity, race, mode of transmission, and family history) were obtained through patient interviews, paper charts, and the clinics’ and hospitals’ electronic database; laboratory data were derived from the database.

### Laboratory analysis

Serologic, mutation, and biochemical tests were performed at baseline at the time of first consultation (Table 1); no patients were tested thereafter for mutations. The limit of detection of HBV DNA quantification is 100 IU/ml. The INNO-LiPA assay had been performed to identify nucleotide polymorphisms at nt1762 and nt1764 in the basal CP region and at codon 28 in the PC region, down to the 4 % level.

**Table 1:** Laboratory test with their respective methods used

Laboratory test	Methodology used
HCV, HDV, HIV	Immunoassays
ALT, AST, bilirubin	Spectrophotometry
INR	Photo-optical clot detection
AFP	Liquid-phase binding assay system
Hepatitis B serologies	Immunoassays
HBV genotype	Genotype specific primers or use of the line probe assay (INNO-LiPA assay v3, Innogenetics NV, Gent, Belgium)
HBV DNA quantification	Roche real time Taqman PCR assay
PC and BCP	INNO-LiPA assay

*HCV* hepatitis C virus, *HDV* hepatitis delta virus, *HIV* human immunodeficiency virus, *HBsAg* HBe antigen, anti-HBe antigen, *INR* international normalized ratio, *AFP* alpha fetoprotein

### Statistical analysis

Relationships between categorical variables were analyzed with Pearson’s chi-square test; Fisher’s exact test was used in cases where an expected cell count was below 5. Continuous outcomes were analyzed with Welch’s *t* test. Because of the censored nature of HBV DNA quantity, we report significant differences among groups based on a parametric survival model, assuming that the log_10_ of HBV DNA quantity is normally distributed. Logistic regression was used to relate continuous and categorical covariates to binary outcomes. With all analyses, a *p* value below 0.05 was considered to indicate a statistically significant result.

## Results

At the time of presentation to the clinic, 39 % (*n* = 459) of patients were nucleos(t)ide analogue treatment-naïve. Table 2 outlines the details of treatment history at or before the time of analysis. The patient population consisted of 64.7 % males. Mean age at the time of presentation was 49.7 (range 15–88 years). Asians (*n* = 684, 57.7 %) and Caucasians (*n* = 361, 30 %) were the dominant races. Non-Hispanic whites (*n* = 322, 27 %) and Chinese (*n* = 216, 18 %) constituted the major ethnic groups. Most patients were identified as infected only with HBV (HBV-monoinfected; no coinfection with HIV, HCV, and/or HDV). However, coinfection status was not known in all patients: of 816 patients with known status, 5.4 % were HIV-infected; of 880 with known status, 9.5 % were HCV-infected; of 410 with known status, 2.9 % were HDV-infected. Complete demographics are outlined in Table 3. The mean MELD score in patients with known cirrhosis was 11.5 (range 6–40) at the time of initial presentation, and 115 (9.7 %) patients had received a liver transplant at the time of their blood sampling. In the 423 patients (35.6 %) with genotype testing, the predominant genotypes were C (30.5 %), A (23.4 %), B (21.5 %) and D (7.8 %); the mutational profile is shown in Table 4. There was a significant difference between genotypes in the prevalence of any mutation versus none (*p* = 0.0426).

**Table 2:** Treatment history of the study population

Parameter	Patients (*n*)	Percent of patients (%)
Treatment-naive	459	38.70
History of treatment with only a single agent		
Lamivudine	102	8.60
Adefovir	20	1.69
Entecavir	161	13.58
Tenofovir	6	0.51
Telbivudine	1	0.08
History of treatment with more than one agent^a^		
Lamivudine and adefovir	6	0.51
Lamivudine and entecavir	7	0.59
Lamivudine and tenofovir	14	1.18
Entecavir and adefovir	6	0.51
Entecavir and tenofovir	1	0.08
Lamivudine, adefovir and entecavir	3	0.25
Unknown	400	33.73

^a^Treatment with these agents either sequentially or in combination

**Table 3:** Demographic characteristics of patients infected with chronic hepatitis B, *N* = 1,186

Characteristic	Value
Age	
Mean (SD)	49.74 (13.54)
Sex	
Female	414 (34.91 %)
Male	767 (64.67 %)
Missing	5 (0.42 %)
FH of HCC	
Neg	792 (66.78 %)
Pos	99 (8.35 %)
Missing	295 (24.87 %)
Race	
Asian	684 (57.67 %)
Caucasian	361 (30.44 %)
Missing	91 (7.67 %)
Black	25 (2.11 %)
Indian subcont.	13 (1.10 %)
Mideastern	6 (0.51 %)
Pacific Island	4 (0.34 %)
Hispanic	2 (0.17 %)
Mode of transmission	
Vertical/childhood	621 (52.36 %)
Missing	363 (30.61 %)
Sexual	88 (7.42 %)
IVDU	37 (3.12 %)
BT	27 (2.28 %)
Needle	24 (2.02 %)
Vert/BT	6 (0.51 %)
IVDU/sexual	4 (0.34 %)
Vert/needle	2 (0.17 %)
Sexual/vertical	2 (0.17 %)
Needle/BT	2 (0.17 %)
IVDU	2 (0.17 %)
Homosexual	2 (0.17 %)
Travel	1 (0.08 %)
Transplant	1 (0.08 %)
Needle/sexual	1 (0.08 %)
IVDU/homosexual	1 (0.08 %)
IVDU/BT	1 (0.08 %)
BT/childhood	1 (0.08 %)

*FH of HCC* family history of hepatocellular carcinoma, *Neg* negative, *Pos* positive, *Indian subcont.* Indian sub-continent, *Vert* vertical, *IVDU* intravenous drug use, *BT* blood transfusion

**Table 4:** Mutational profile of each HBV genotype

HBV genotype	No mutation	CP only	PC only	PC/CP mix	Any mutation
A	13 (43 %)	3 (10 %)	10 (33 %)	4 (13 %)	17 (57 %)
B	8 (17 %)	3 (6 %)	20 (43 %)	16 (34 %)	39 (83 %)
C	11 (23 %)	7 (14 %)	25 (51 %)	6 (12 %)	38 (78 %)
D	1 (11 %)	0 (0 %)	6 (67 %)	2 (22 %)	8 (89 %)

There is a significant difference between genotypes in the prevalence of any mutation versus none (*p* = 0.0426). Data is presented as counts (percentages)

### Prevalence of mutations

**Fig. 1:**
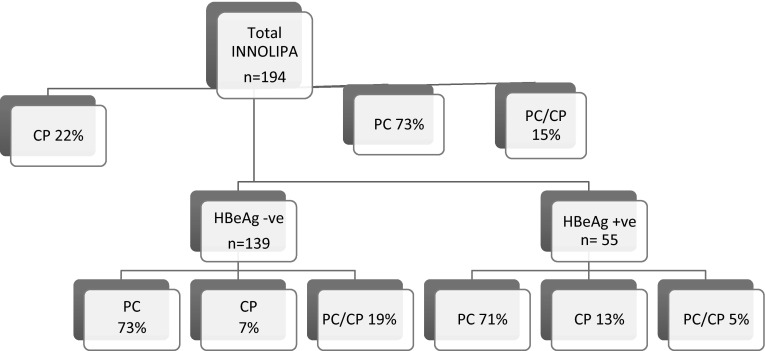
Distribution of PC and/or CP mutations with respect to HBeAg-positive and HBeAg-negative patients. *HBeAg +ve* HBeAg positive, *HBeAg –ve* HbeAg negative, *PC* precore mutations only, *CP* core promoter mutations only, *PC/CP* both precore and core promoter mutations

Of the total population (*n* = 1,186), 926 patients were tested for HBeAg status. Of the patients tested, 37 % (*n* = 344) were HBeAg-positive and 63 % (*n* = 582) were HBeAg-negative. The INNO-LiPA test to check for PC and CP mutations was performed in 194 patients; details of prevalence of mutations in HBeAg-positive and HBeAg-negative patients are shown in Fig. 1. In the overall population, the difference in the prevalence of PC mutations, CP mutations or both in HBeAg-positive patients (56 %) and HBeAg-negative patients (89 %) was statistically significant (*p* < 0.001). When considered separately, there was also a significant difference in the prevalence of PC and/or CP mutations between treatment-naïve HBeAg-positive and HBeAg-negative patients (*n* = 103 and 83 % had PC and/or CP mutations; *p* < 0.001), although not in the treatment-experienced (*n* = 72, 77 % PC and/or CP mutations; *p* = 0.193). There was no statistically significant difference in the distribution of the confounding variables of race, gender, or presence of coinfection with HIV, HCV, or HDV in patients with or without these mutations. However, older age was associated with a higher prevalence of PC mutations (*p* = 0.013) in the whole population. The difference in the prevalence of PC mutations in HBeAg-negative and HBeAg-positive patients was statistically significant when considering the whole sample (*p* < 0.001) and after controlling for age in a logistic regression done on the entire sample (*p* < 0.001) and the treatment-naïve subpopulation (*p* < 0.001), but not in the treatment-experienced or monoinfected subpopulations.

## ALT levels with and without mutations

**Fig. 2:**
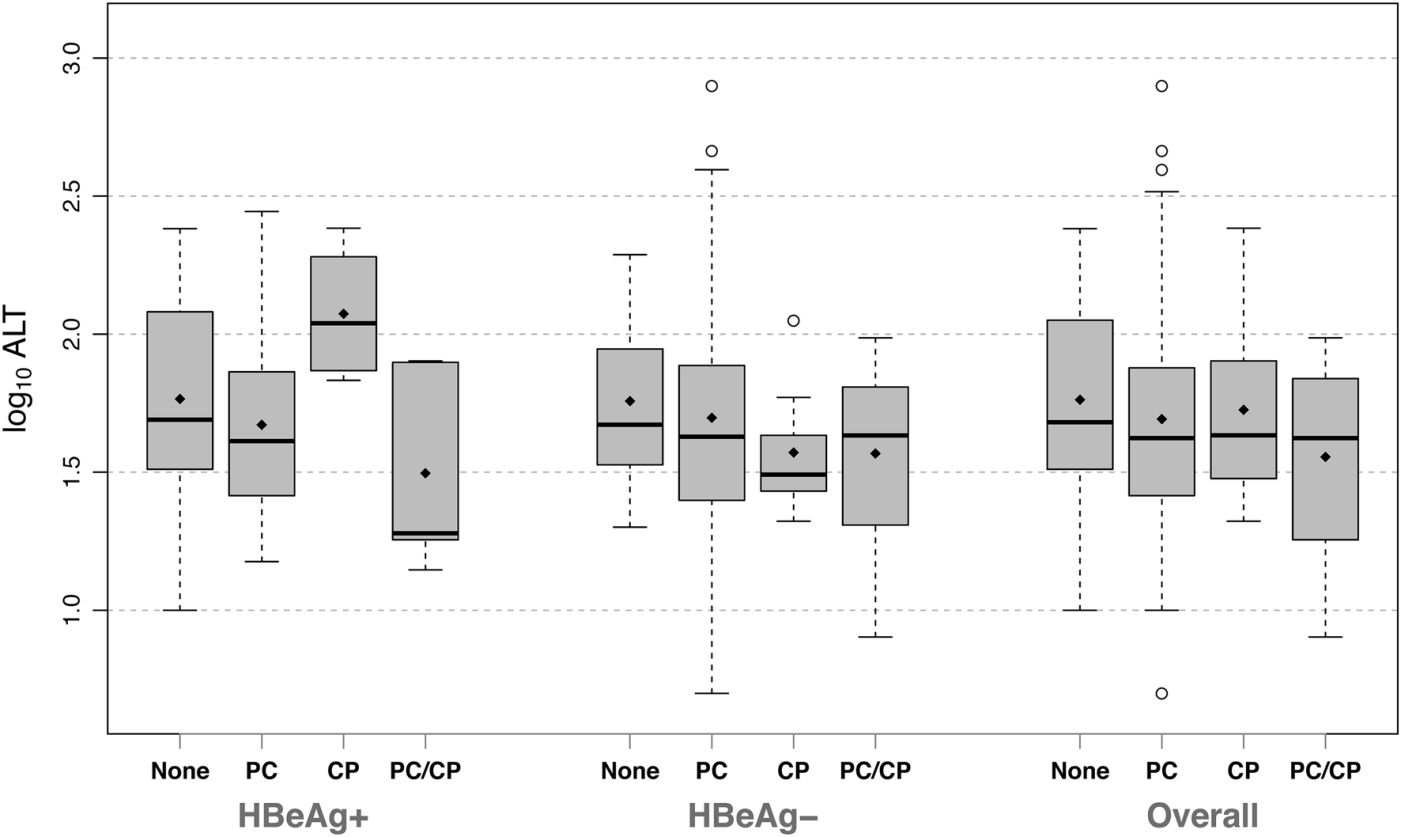
ALT levels relative to precore and core promoter mutations. The *diamonds in each boxplot* represent the mean for that group, while the *solid line* represents the median. Outliers are defined as being beyond 1.5 times the interquartile range (IQR) from the median, and are represented by *empty circles*. *PC* precore mutations only, *CP* core promoter mutations only, *PC-CP* both precore and core promoter mutations, *HBeAg+* hepatitis B e antigen positive, *HBeAg−* hepatitis B e antigen negative

The mean log_10_ ALT for the whole population was 1.68 (range 0.47–3.64) as determined at the time of first consultation. There was a significant difference in the mean log_10_ ALT of patients with both PC and CP mutations (1.56, SD = 0.30) when compared to the whole population of patients without these mutations (1.76, SD = 0.35, *p* = 0.011) (Fig. 2). We found no significant differences in mean log_10_ ALT when focusing on the treatment-experienced and monoinfected population. However, in the treatment-naïve group, we found a significant difference between patients with both PC and CP mutations (1.54, SD = 0.29) when compared to patients with no mutation (1.78, SD = 0.34, *p* = 0.41). When controlled for genotype in a linear model, we did not find a significant difference in log_10_ ALT between any mutation groups.

### Association of various ALT levels with liver disease

To see the association of various levels of ALT with cirrhosis and HCC, the study population was divided into four sub-groups according to the ALT level at the first time of diagnosis. As shown in Table 5, these included patients with ALT level ≤0.5 × ULN (group 1), 0.5–1 × ULN (group 2), 1–2 × ULN (group 3) and >2 × ULN (group 4). Our analysis showed significantly higher incidence of cirrhosis in group 3 (OR 3.514, *p* = 0.013) and group 4 (OR 2.994, *p* = 0.031) when compared to group 1. We did not find a significant difference between any ALT groups in the treatment-experienced and monoinfected subpopulations. However, we did find a significant difference between group 4 and group 1 in the treatment-naïve group (OR 8.110, *p* = 0.0491). When controlling for HBV genotype in a logistic regression model, we did not find a significant difference in the incidence of cirrhosis between any ALT groups. We also found a mathematical increase in the incidence of HCC with the increase in ALT level (5.6 % in group 1, 8.6 % in group 2, 14.7 % in group 3 and 15.1 % in group 4) although these increases were not found to be statistically significant when compared to group 1 (Table 5). We did not find any significant differences in the incidence of HCC across ALT groups when controlling for HBV genotype or within the treatment-naïve, treatment-experienced, and monoinfected subgroups.

**Table 5:** Prevalence of hepatocellular carcinoma (HCC) and cirrhosis according to ALT level

ALT group (range)	HCC		Cirrhosis	
	Patients (%)	*p* value	Patients (%)	*p* value
Group 1 (0–14)	5.6	_	16.7	–
Group 2 (15–29)	8.6	0.460	28.6	0.182
Group 3 (30–59)	14.7	0.081	41.3	0.013*
Group 4 (≥60)	15.1	0.071	37.5	0.031*

The *p* values correspond to a logistic regression with group 1 treated as the reference group

* Statistically significant

### Effect of mutations on HBV viral load

**Fig. 3:**
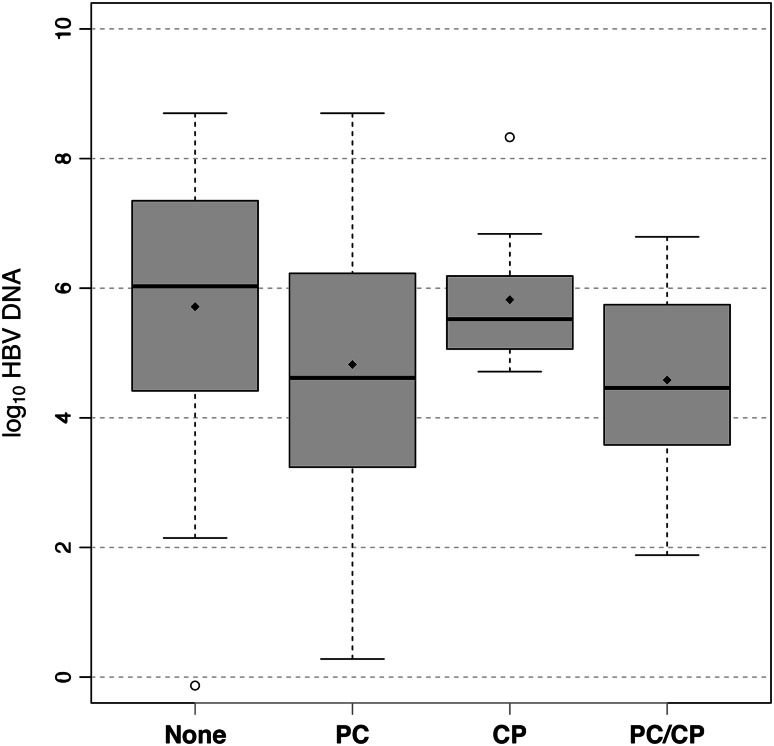
Effect of mutations on HBV viral levels. For the sake of visualization, right-censored values are left at their lower limit. The *diamonds in each boxplot* represent the mean for that group, while the *solid line* represents the median. Outliers are defined as being beyond 1.5 times the interquartile range (IQR) from the median, and are represented by *empty circles*. *PC* precore mutations only, *CP* core promoter mutations only, *PC-CP* both precore and core promoter mutations

The HBV DNA levels were measured at the time of INNO-LiPA testing in those patients who had INNO-LiPA testing; the DNA levels stated were at the time of first consultation if the INNO-LiPA test was not performed. The effect of various mutations on the log_10_ HBV DNA was evaluated. The mean log_10_ HBV DNA of patients without any mutations was 5.71 (SD = 2.28). In the overall population, when compared with patients without mutations, the mean log_10_ HBV DNA was significantly lower in patients with PC mutations only (4.82; SD = 1.98, *p* = 0.019) (Fig. 3); those with CP mutations only (5.82, SD = 1.20) and patients with both PC and CP mutations (4.58; SD = 1.37) were not found to have significantly different mean log_10_ HBV DNA (*p* = 0.895 and *p* = 0.074, respectively). PC mutation only was also found to be significantly associated with lower HBV DNA in a survival regression model controlled for age (*p* = 0.029). In separate analyses, no significant differences were found in log_10_ HBV DNA across mutations in patients infected only with HBV (excluding patients coinfected with HIV, HCV, or HDV) or in treatment-experienced patients. However, in the treatment-naïve group, we found a significant difference between patients with PC mutations only (4.65, SD = 1.77, *p* < 0.001) and those with both PC and CP mutations (3.87, SD = 1.39, *p* < 0.001) when compared to subjects without any mutations (6.18, SD = 1.90).

### Incidence of cirrhosis

**Fig. 4:**
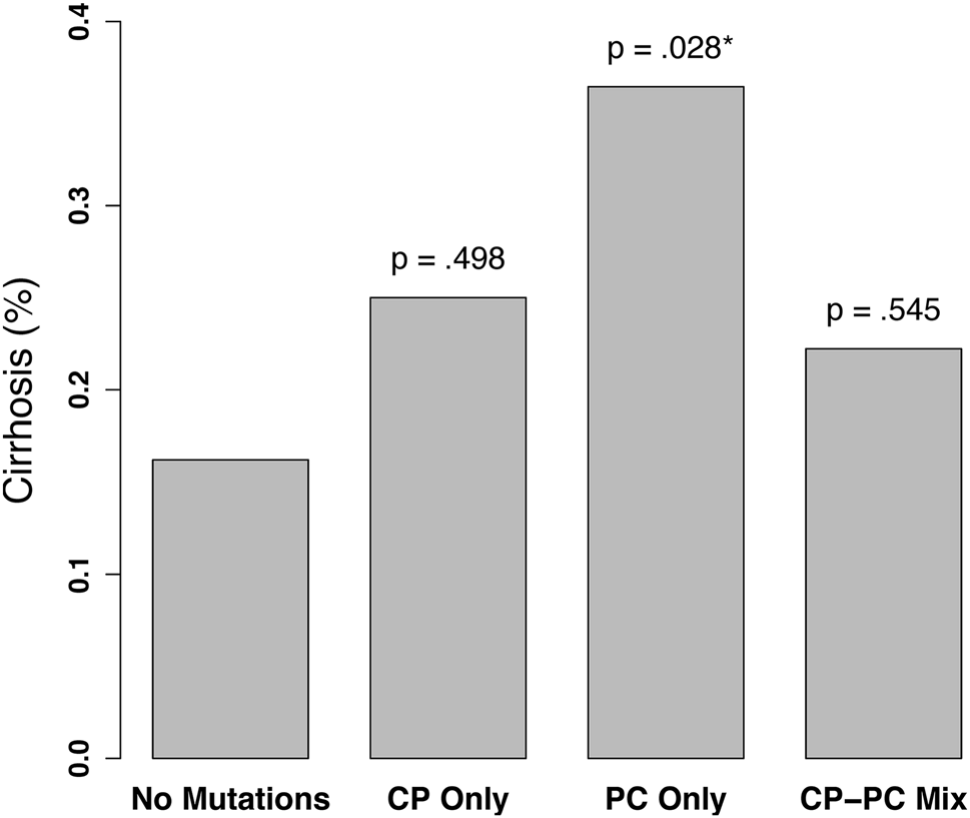
Effect of mutations on incidence of cirrhosis. Each *p* value represents the comparison of that group with the no mutations group, computed from a logistic regression. *PC* precore mutations only, *CP* core promoter mutations only, *PC-CP* both precore and core promoter mutations

The incidence of cirrhosis (histological or clinical) was also evaluated in the study population. We found that 37 % (268 patients) of the 718 CHB patients who were tested had cirrhosis, with the incidence similar in HBeAg-positive and HBeAg-negative populations (32 % and 37 %, respectively). Univariate analysis for the incidence of cirrhosis showed older age (*p* < 0.001), male sex (*p* < 0.001), Caucasian race (*p* < 0.001), presence of HDV antibody (*p* < 0.001), presence of HCV antibody (*p* = 0.005), and PC only mutation (*p* = 0.028) to be associated with higher incidence of cirrhosis (Fig. 4); there was a lower rate of cirrhosis in patients with genotype B and C relative to patients with genotype A (*p* = 0.002 and *p* = 0.007, respectively). Those covariates whose *p* values were below 0.05 were included in a multiple logistic regression. However, the multiple logistic regression model did not confirm any covariates to be associated with cirrhosis. We also included both genotype and mutation in a separate multivariate model, and confirmed the higher rate of cirrhosis in subjects with PC mutations when compared to subjects with no mutation when controlled for variations across HBV genotype (*p* = 0.020, OR 5.435). When testing for an interaction between any of the above covariates and treatment history, we only find a significant difference in the effect of age between treatment-naïve and -experienced patients, with the odds of cirrhosis increasing at a slower rate as a function of age in the treatment-naive group (*p* = 0.0298). We do not find any significant differences between covariates effects between monoinfected and coinfected patients.

### Incidence of hepatocellular carcinoma

There were 129 patients found to have HCC in our study population. The mean alpha-fetoprotein (AFP) was 1,737 (range 1.1–53,727) in patients with HCC. Similarly to another recent study [21], we found a significantly higher incidence of HCC in patients with HBeAg-negative disease (*n* = 84, 15 %) as compared to patients with HBeAg-positive status (*n* = 19, 6 %, *p* < 0.001). We also found a significantly higher incidence of HCC in HBeAg-negative patients as compared to HBeAg-positive in both the treatment-naïve and -experienced groups (13 vs. 4 %, *p* = 0.020 and 21 vs. 9 %, *p* = 0.007, respectively), but not in the HBV-monoinfected group (6 vs. 4 %, *p* = 0.75). A total of 16 patients with HCC underwent INNOLiPA testing; of these, ten tested positive for PC mutation only, two tested positive for both PC and CP mutations, and four tested negative for both PC and CP mutations. There were no significant differences in the incidence of HCC in relation to the presence of CP only, PC only, or both PC and CP mutations when looking at the entire study population, treatment-experienced patients, treatment-naïve patients, or monoinfected patients (excluding coinfected patients), nor was there a significant difference in the distribution of HBV genotype in patients with and without HCC in any of these subgroups.

## Discussion

We confirmed the occurrence of CP and/or PC mutations in the viral populations of a substantial percentage of HBeAg+ patients. In previous US studies, the incidence of PC mutations (only) in HBeAg-positive patients has been reported as ranging from 9 % [4] to 28 % [5], while in our population, of the 55 HBeAg-positive patients who underwent iNNOLipa testing, the incidence was 71 %. In our study, in the whole population of patients who underwent iNNOLipa testing (*n* = 194), the overall prevalence of PC mutations was 73 %. The much lower overall 27 % PC prevalence reported by Chu et al. [4] may be explained by the selection bias of only including treatment-naïve patients while our study drew from what may be a much more representative community population that included both treatment-experienced and -naïve patients.

The 13 % incidence of CP mutations in our HBeAg-positive patient population is lower compared to previous US studies which have reported a range from 28 % [5] to 36 % [4]. We found a higher prevalence (9 %) of HBeAg-positive patients harboring both PC and CP mutations compared to the prevalence of 4 % [4] previously reported. In genotypes B and C, the previously reported prevalence of PC mutations was 34 and 7 %, respectively [5], substantially lower than the prevalence we found in these genotypes (B, 43 % and C, 51 %). We found higher prevalence of PC mutations with older age, in contrast to Vutien et al. [5] who reported higher prevalence of CP mutations with age >40 years, a finding that may be partially explained by a possible selection bias created by their inclusion of almost entirely only Asian American patients with genotypes B and C.

We also found a statistically significant difference in the prevalence of PC mutations only in HBeAg-negative and HBeAg-positive patients when considering the whole sample and the treatment-naïve subpopulation. We did not find the association between the prevalence of these variants and ethnicity and place of birth previously reported in a study of treatment-naïve patients from tertiary care centers [4], possibly due to a change in the prevalence of these mutations as the CHB population ages and the virus undergoes mutations with longer duration of disease. In addition, our population consisted of an equal proportion of treatment-naïve and -experienced patients from all ethnic backgrounds and from both tertiary care centers and community clinics and thus may be more representative of the prevalence of HBV variants in the United States.

In previous studies there have been varying reports on the association of mutations with the severity of liver disease. We found a higher rate of cirrhosis in patients with only PC mutations when compared to patients with no mutations when controlled for variations across HBV genotypes. Chu et al. [4] reported that patients with CP mutations with or without PC mutations were more likely to have decompensated cirrhosis. Since the lower number of patients in some of our sub-populations may have influenced these results, further research will be needed to elaborate on these findings. The association of PC and CP mutations with HBV viral load has also been unclear in both HBeAg-negative and -positive patients. In our study we found significantly lower levels of HBV DNA in patients with PC mutations only in comparison to patients with no mutations in both a univariate survival regression model (mutation only) and in a model controlled for age. We found significantly lower levels of DNA in patients with only PC mutations and patients with both PC and CP mutations in the treatment-naïve population, a difference that was not observed in treatment-experienced patients.

Multiple studies have reported an association between CP mutations and an increased risk of HCC [9, 10, 12, 22]. A study by Yang et al. [13] found this association, combined with an apparent protective effect of PC mutation, with a hazard ratio of developing HCC of 0.34 for precore G1896A versus wild type. In our study, there were no significant differences in the incidence of HCC in relation to the presence of PC only or CP only mutations, or the combination of both PC and CP mutations. Although we did not find a clear association of PC mutations with either the development of or protection against HCC, it is important to recognize the limits of this study design. In addition, there continues to be a controversy about whether CP and PC mutations are dominant factors in the development of HCC. The presence of these mutations may be confounded by age, genotype, and other variables. Ultimately, the best approach to taking the presence of these mutations into account in terms of predicting HCC risk may be to place them in the type of scoring system proposed by UCLA researchers in which CP mutation is only one of multiple factors previously identified as independent risk factors which are assigned numerical scores that are weighted based on expert opinion in order to yield a risk impact score that may be used to make treatment decisions [23, 24].

Higher levels of ALT have been associated with advanced liver fibrosis or cirrhosis, and have served as a predictor for the development of complications of CHB. Kim et al. [25] reported that there is a positive association between serum aminotransferase concentration, even when it is within the current normal range (35–40 IU/l), and mortality from liver disease. A study by Yuen et al. [26] showed that ALT levels of 0.5–2 × ULN (ULN: 53 U/l for males and 31 U/l for females) are associated with an increased risk of developing complications (including ascites, esophageal varices, encephalopathy and HCC) compared with patients with ALT levels <0.5 × ULN. In our study, with the upper limit of normal defined as 30 for both males and females, patients with ALT 0.5–1 × ULN were not found to be at increased risk of developing cirrhosis or HCC as compared to patients with ALT <0.5 × ULN. However, we found a significantly higher incidence of cirrhosis in patients with ALT level 1–2 × ULN (OR 3.514, *p* = 0.013) and patients with ALT level >2 × ULN (OR 2.994, *p* = 0.031). If confirmed, this observation could put in question the guidelines made by liver societies worldwide in which treatment is only recommended with ALT > 2 × ULN.

Our findings of the presence of significantly lower log_10_ ALT levels in association with patients who have both PC and CP mutations may serve as a basis for further studies to evaluate the duration of treatment of such patients. This could be an important observation, especially in regard to treatment duration of patients with HBeAg-positive and -negative disease. The exact mechanism of this difference is not entirely clear but the likely explanation could be that our patient population included both treatment-naïve patients and those who had been treated with nucleos(t)ide analogues. This may show that development of combined PC and CP mutations, whether in the natural history of CHB or during antiviral treatment, carries a better prognosis. A limitation of our study is that, given the fluctuating nature of CHB, a cross-sectional approach may over- or underestimate the conclusions drawn. Thus, a longitudinal study will be needed to confirm our findings.

Treatment guidelines from such major liver societies as the American Association for the Study of Liver Diseases (AASLD), the European Association for the Study of the Liver (EASL) and the Asian Pacific Association for the Study of the Liver (APASL) take into account ALT levels as one of the indicators to start therapy [27–29]. According to these guidelines, both HBeAg-positive and -negative patients with ALT ≤2 × ULN can be observed without treatment. Our study showed an increased incidence of cirrhosis in patients with ALT level >1 × ULN. Further studies are needed to clarify the ALT level that should be used as an indicator for beginning therapy.

In addition, one of the criteria for stopping treatment is based on HBeAg seroconversion in HBeAg-positive CHB. The criteria for stopping treatment are unclear in HBeAg-negative CHB. And this raises a major question: when to stop therapy in a patient with HBeAg+ disease who also harbors both PC and CP mutations. Some patients are committed to therapy with antivirals for life if they are not able to attain the predefined end points such as sustained virologic response (undetectable HBV DNA) and biochemical response (normal ALT). The longer duration of treatment with antiviral agents puts patients at risk for the development of resistant HBV and medication-related side effects. In our study, we showed that PC and CP mutations may be associated with milder liver disease in some patients, with lower ALT levels in those with both PC and CP mutations, and lower HBV DNA levels in those with PC mutations only. Our cross-sectional study may serve as a basis for longitudinal studies, which could elucidate this observation and help delineate treatment need and duration in patients with these HBV variants.

## Abbreviations

CHB: Chronic hepatitis B
HBeAg: Hepatitis B e antigen
CP: Core promoter mutation
PC: Precore mutation
HBV: Hepatitis B virus
anti-HBe: Hepatitis B e antibody
HCC: Hepatocellular carcinoma
HBsAg: Hepatitis B surface antigen
Anti-HBc: Hepatitis B core antibody
IgG: Immunoglobulin G
CPMC: California Pacific Medical Center
HCV: Hepatitis C virus
HDV: Delta hepatitis virus
HIV: Human immunodeficiency virus
AFP: Alpha-fetoprotein
AASLD: American Association for the Study of Liver Diseases
EASL: European Association for the Study of the Liver
APASL: Asian Pacific Association for the Study of the Liver
